# Behaviors of Turkish pregnant women towards gestational diabetes screening

**DOI:** 10.12669/pjms.37.5.4176

**Published:** 2021

**Authors:** Huseyin Aydogmus, Serpil Aydogmus, Halil Ibrahim Tiras, Zeynep Cankaya

**Affiliations:** 1 Huseyin Aydogmus, M.D. Department of Gynecology and Obstetrics, Izmir Katip Celebi University Ataturk Research and Training Hospital, Izmir, Turkey; 2 Serpil Aydogmus, M.D. Department of Gynecology and Obstetrics, Izmir Katip Celebi University Ataturk Research and Training Hospital, Izmir, Turkey; 3 Halil Ibrahim Tiras, M.D. Department of Gynecology and Obstetrics, Izmir Katip Celebi University Ataturk Research and Training Hospital, Izmir, Turkey; 4 Zeynep Cankaya, M.D. Department of Gynecology and Obstetrics, Izmir Katip Celebi University Ataturk Research and Training Hospital, Izmir, Turkey

**Keywords:** Gestational Diabetes, Oral Glucose Tolerance Test, Perinatal Results

## Abstract

**Objectives:**

Although gestational diabetes is the most common metabolic disease in pregnancy some pregnant women still refuse to undergo oral glucose tolerance test (OGTT). The purpose of this study was to evaluate the behavior of pregnant women undergoing OGTT, and to compare perinatal results between women who undergo and refuse OGTT.

**Methods:**

This retrospective cohort study was performed by evaluating the data of Izmir Katip Celebi University Gynecology and Obstetrics outpatient clinic between 2012-2017. Data of 2079 pregnant were evaluated retrospectively. Among 373 women who refused OGTT were evaluated as the study group, while remaining 1706 women who underwent OGTT were considered as the control group. The groups were compared with regard to perinatal results.

**Results:**

Sixty-two point four percent of the group who refused OGTT had a C-section, while 56.3% of the control group had a C-section (p<0.05). Intrauterine growth retardation, fetal distress, amniotic fluid pathologies, macrosomia, gestational hypertension and perinatal death were slightly higher in pregnant women who did not undergo OGTT compared to the control group, however, the difference was not statistically significant.

**Conclusion:**

Maternal complications and poor pregnancy results were found slightly higher in pregnant women who refused OGTT. These results might be explained by assuring glycemic control in pregnant women who refused OGTT by a series of fasting and postprandial blood sugar measurements in our center.

## INTRODUCTION

Gestational diabetes is defined as a type of glucose intolerance that is first recognized in pregnancy. The prevalence of gestational diabetes (GDM) has been reported to be 14% across the globe and approximately 12.6% in Europe. Incidence of GDM has shown a progressive increase in recent years.^1^,^2^

Similar to other Mediterranean countries, Turkish society is a community with high GDM prevalence. Interestingly, a study performed in Germany, which is a country with high Turkish population, Turkish women living in the same area with German women were reported to have higher GDM prevalence (13.8% versus 18.3%).^3^ A recent multi-center study performed on Turkish population found that the prevalence was 16.2% across Turkey without any difference among regions.^4^

GDM has negative maternal and fetal consequences.^5^ Various pregnancy complications such as gestational hypertension (GHT), macrosomia, C-section, preterm labor, and intrauterine fetal death (IUMF) are reported to increase neonatal complication risk by 1.2-1.6 times in GDM.^6^ Despite the risks posed by uncontrolled GDM to the course of pregnancy and the newborn, there are confusing reports in the media about OGTT. Some of public web resources indicate that ready-to-use glucose solution used in OGTT or glucose challenge test (GCT) has harmful effects on the fetus. In 2014, a Turkish academician who is not an obstetrician made a statement on media in which he claimed OGTT may have harmful effects on the fetus, and consequently, refusal of OGTT increased remarkably among pregnant women admitted to our hospital. Recently, similar observations have been reported from a center in a different region of Turkey.^7^ The purpose of this study is to evaluate the behavior of pregnant women with regard to taking OGTT, and to compare perinatal results in women who did and did not undergo OGTT.

## METHODS

This retrospective cohort study reviewed data of 3164 women admitted to Izmir Katip Celebi University Clinic of Gynecology and Obstetrics for antenatal follow-up and/or labor between 2012-2017. Demographic and clinical data of pregnant women were obtained from the hospital’s electronic database system. All singleton pregnancies without known chronic systemic diseases and pregestational diabetes were included in the study. The study was approved by Izmir Katip Celebi University local ethics committee (2018/182). Replications, and women whose perinatal monitoring and/or birth data could not be accessed were excluded from the study. Among a total of 2079 pregnant women included in the study, 373 women who refused OGTT were evaluated as the study group, while remaining 1706 women were considered as the control group. Women were diagnosed with GDM upon having at least one of the following values according to ADA/IADPSG criteria: FBG ≥92 mg/dL, ≥180 mg/dL, Hour-2 ≥153 mg/dL; while according to Carpenter-Coustan criteria GDM diagnosis was made in the presence of at least two positives among FBG ≥105mg/dL Hour-1, ≥190mg/dL, Hour-2 ≥165mg/dL, Hour -3 ≥145 mg/dL. SGA and fetal macrosomia were defined as a birth weight below the 10th percentile for gestational age and gender, and as a birth weight above 4000 grams, respectively. Preterm labor was defined as labor before the completion of week 37, gestational hypertension as systolic blood pressure ≥140 mmHg and/or diastolic blood pressure ≥90 mmHg after week 20, and preeclampsia as concomitant hypertension and any signs of end organ damage.

The data was evaluated in IBM SPSS Statistics 22.0 (IBM Corp., Armonk, New York, USA) statistical package program. Descriptive statistics were expressed as number (n), percentage (%), mean ± standard deviation (x ± SD), and median values. Normal distribution of continuous numeric variables was assessed with Shapiro Wilk normality test. Independent sample T-test or Mann-Whitney test was used for the intergroup comparisons of continuous data. Fisher’s chi-square exact test was used for comparing the groups according to categorical variables. P-values <0.05 were assumed to be statistically significant.

## RESULTS

Groups were similar with regard to gravidity, parity, gestational age at birth, biometric values of the newborn, and Apgar scores (Table-I). Total rate of GDM was 14.8% in our study group. In both groups population whom were diagnosed GDM according to ADA/IADPSG criteria and the rate of pregnant women treated with insulin were similar. C-sections consisted of 62.4% of the group who did not take OGTT and 56.3% of the control group (p<0.05). Placental abnormalities, preterm labor, dystocia and maternal complications were similar in both groups. Fetal abnormalities were detected in 1.1% of the group who underwent OGTT, and 0.6% of the group who refused OGTT. Intrauterine growth restriction, fetal distress, amniotic fluid pathologies, macrosomia, gestational hypertension and perinatal death were slightly higher in pregnant women who did not undergo OGTT compared to control group. However, none of these differences between the groups were statistically significant (Table-II).

**Table-I:** General characteristics of the groups.

	Gravidity	Parity	Gestational Age (weeks)	Delivery Route (C-Section%)	C-Section Reason (%)	Gender (Male%)	Height (cm)	Birth Weight (Grams)	Head Circumference (cm)	APGAR 5.min
Refused-OGTT	3 (2-4)	1 (1-2)	39.6	62.4	36.6	53.1	49.8±2.4	3299.7±495.7	34.6±1.5	9.64±1.55
Underwent-OGTT	2 (2-3)	1 (1-2)	39.4	56.3	34.4	52.5	49.9±2.5	3293.7±521.2	34.4±1.6	9.67±1.55
P	0.02	0.03	0.07	0.02	0.67	0.82	0.70	0.84	0.12	0.11

**Table-II:** Comparison of groups with regard to perinatal results.

	Underwent OGTT (n=1706)		Refused OGTT (n=373)		P
	n	(%)	n	(%)	0.45
Amniotic fluid pathologies	5	1.3	49	2.9	
Placental abnormalities	5	1.3	22	1.3	
Preterm labor	0	0	6	0.4	
Fetal distress	15	4.0	84	4.9	
Dystocia	51	13.7	190	11.1	
Perinatal death	2	0.5	12	0.7	
Fetal abnormalities	4	1.1	11	0.6	
Macrosomia	10	2.7	51	3.0	
Gestational hypertension	7	1.9	35	2.1	
IUGR	2	0.5	35	2.1	
Maternal complications	9	2.4	39	2.3	

Total	110	29.5	541	31.7

Assessment of the pregnant women’s compliance behavior about undergoing OGTT, pregnant women were divided in two groups by assuming the year 2014 as cutoff point, the year publications on so-called harmful effects of OGTT were released into the media. Comparison of the approach to OGTT between 325 pregnant women admitted in 2014 and earlier, and 1754 pregnant women admitted in 2015 and later, refusal of OGTT rose from 4.9% to 20.3% (RR: -0.15; 95% CI -0.184 -0.124) (P = 0.00).

## DISCUSSION

The current study revealed that the rate of C-sections in pregnant women who refused OGTT was significantly higher than women who underwent OGTT. Although the incidence of complications such as intrauterine growth restriction, fetal distress, amniotic fluid pathologies, macrosomia, gestational hypertension (GHT) and perinatal death were slightly higher in women who refused OGTT, the difference was not statistically significant. To the best of our knowledge, there is no study comparing the perinatal results of pregnant women who had OGTT and those who refused this test in the literature

The prevalence of GDM, the most common medical complication in pregnancy, has been rising over time.^8^ Hyperglycemia in pregnancy is associated with short and long-term complications for the mother, the fetus, and the newborn.^9^,^10^ Increased prevalence and its concomitant important complications has led to the development of GDM screening programs, however screening is still controversial. No consensus has yet been established on the exact timing, threshold values, one- or two-step scans and whether to perform selective or universal screening.^5^,^11^ A recent discussion has suggested to change the current threshold values and that GDM incidence might increase if fasting and postprandial serum glucose levels accepted in the HAPO study, recognized as the reference point, are to be used in our daily practice.^12^

We think that refusal of OGTT in pregnant women increased significantly with the reportrs in the media. Even though current medical guidelines declare that OGTT has no negative maternal or fetal effects, the considerable increase in refusing OGTT in Turkey since 2014 was due to negative media reports. We have determined that refusal of OGTT among pregnant women in our region increased from 4.9% to 20.3% between the specified dates. Similar results have been obtained in a study performed in a different region of Turkey.^7^ Another Turkish study has reported a significantly positive correlation between a positive approach to OGTT and educational level as well as informing the patient sufficiently. Forty-six percent of women who refused OGTT have stated their concerns about the test being harmful either for themselves or the fetus. Thirty-two percent of the pregnant women in that study reported the source of their mis-information to be the TV or radio; likewise, 40% of pregnant women reported the same in a similar study from another country.^13^,^14^ In a study from another city of our country, it was reported that 49% of pregnant woman refused OGTT, and more than half of them thought the test was harmful for them or their baby. Interestingly, it was reported that there was no relationship between OGTT refusal and educational level.^15^

We found that perinatal complications, except of the higher cesarean rates, were not statistically significant in the OGTT refusal group. This may be attributed to the fact that FBG or random blood glucose measurement is performed in the first trimester within the universal screening principle in our clinic. We closely follow the patients whose FBG were measured more than 92 mg/dL in the first antenatal visit and we also measure fasting and postprandial blood glucose once at least one time in the third trimester for all pregnant women who do not accept OGTT. Although the cut off level of FPG is controversial in the literature, there are increasing number of studies reporting that FPG measurement in the first trimester is an effective and reproducible test for the prediction of GDM development and perinatal complications.^16^-^20^ So, it might be speculated that those procedures, even in the absence of OGTT, allowed the diagnosis of a potential glucose metabolism disorder prior to the emergence of serious complications.

### Strength and weakness of the study

The strength of our study is the high number of patients. On the other hand, the retrospective evaluation of the data constituted the weakness of our study due to limited access to pregnancy results of some subjects, having had to exclude them. Furthermore, the results of our study could not be interpreted in a comparative manner since, to the best of our knowledge, no similar comparative study on the literature exists.

## CONCLUSION

The effect of information provided in media is considerably high on society’s perception of health. In order for screening programs to be successful, compliance of the society should be ensured, and for that, the target group should be informed accurately and sufficiently. Considering the increasing prevalence of gestational diabetes, compliance of pregnant women with screening programs and treatment poses a great importance for public health. Turkish Ministry of Health and national associations of gynecology and obstetrics have been taking the necessary precautions associated with this problem that occurred with the effect of media.

### Authors’ Contribution:

**HA, SA:** Conceived, designed and did statistical analysis & editing of manuscript.

**HIT, ZC:** Did data collection and manuscript writing.

**SA:** Did review and final approval of manuscript.

**H.A**: Responsible and accountable for the accuracy or integrity of the work.
